# Supplementary material for: Evaluating the Impact of Practical Training: A Study on Satisfaction and Drug Knowledge among Pharmacy Students
Source: Pharmacy (Basel). 2024 Apr 16;12(2):69. doi: 10.3390/pharmacy12020069 (PMC11053554; doi:10.3390/pharmacy12020069)
Supplement: Supplementary file 1 [file pharmacy-12-00069-s001.zip › pharmacy-2885753-supplementary.pdf]

Supplementary Material

Table SI. Drug names listed in the questionnaire (categorised by sales order)

Drug name	Sales (million yen)	Quantity (10,000 pieces)
Esomeprazole magnesium hydrate	75998	58200
Sitagliptin phosphate hydrate	71018	52435
Rosuvastatin calcium	67441	104461
Pregabalin	66328	74175
Eldecalcitol	49905	51578
Clopidogrel sulfate	43788	38699
Olmesartan medoxomil	43600	50614
Celecoxib	38202	54350
Lansoprazole	32736	71510
Febuxostat	29158	61745
Telmisartan	28628	36558
Levocetirizine hydrochloride	28357	49101
Ethyl Icosapentate	27999	50214
Silodosin	26342	36171
Candesartan cilexetil	22783	37909
Cilostazol	21111	36624
Olopatadine hydrochloride	20434	55448
Bepotastine besilate	19443	43472
Rebamipide	19281	176231
Pranlukast hydrate	19183	45526
Clarithromycin	18154	36646
Pravastatin sodium	16439	44478
Metformin hydrochloride	16202	156706
Magnesium oxide	16187	303816
Nifedipine	14698	75761
Zolpidem tartrate	13921	39132
Loxoprofen sodium hydrate	11928	117552
Rivaroxaban	54675	11821
Ezetimibe	46864	25291
Tacrolimus hydrate	46065	7325
Vonoprazan fumarate	41848	23035
Memantine hydrochloride	41202	13737
Limaprost alfadex	31466	86426
Galantamine hydrobromide	23456	13008
Vildagliptin	20968	26177
Dutasteride	20714	9836
Escitalopram oxalate	19638	9075

Mirtazapine	18372	10764
Lubiprostone	18130	11254
Aripiprazole	16148	13866
Lamotrigine	15765	11253
Tolvaptan	30423	1968
Enzalutamide	21733	923
Bicalutamide	21499	4550
Dasatinib	20567	258
Osimertinib mesilate	16572	69
Nilotinib hydrochloride hydrate	14279	364
Alectinib hydrochloride	13814	209
Tadalafil	12890	4374
Bosentan hydrate	12407	276

From the NDB's top 100 drug sales data, 50 drugs were randomly selected for the drug name knowledge survey. These are the 50 drugs that were used.
